# Inhibition of cytochrome P450 enhances the nephro- and hepatotoxicity of ochratoxin A

**DOI:** 10.1007/s00204-022-03395-y

**Published:** 2022-10-13

**Authors:** Reham Hassan, Daniela González, Zaynab Hobloss, Lisa Brackhagen, Maiju Myllys, Adrian Friebel, Abdel-latif Seddek, Rosemarie Marchan, Benedikt Cramer, Hans-Ulrich Humpf, Stefan Hoehme, Gisela H. Degen, Jan G. Hengstler, Ahmed Ghallab

**Affiliations:** 1 Leibniz Research Centre for Working Environment and Human Factors, Technical University Dortmund, Ardeystr. 67, 44139 Dortmund, Germany; 2 Department of Forensic Medicine and Toxicology, Faculty of Veterinary Medicine, South Valley University, Qena, 83523 Egypt; 3 Institute of Computer Science and Saxonian Incubator for Clinical Research (SIKT), University of Leipzig, Haertelstraße 16-18, 04107 Leipzig, Germany; 4 Institute of Food Chemistry, Westfälische Wilhelms-Universität Münster, Corrensstr. 45, 48149 Münster, Germany

**Keywords:** Mycotoxins, Detoxification, Bioactivation, Metabolic zonation, Drug metabolism

## Abstract

The mycotoxin ochratoxin A (OTA) is a contaminant in food that causes nephrotoxicity and to a minor degree hepatotoxicity. Recently, we observed that OTA induces liver damage preferentially to the cytochrome P450 (CYP)-expressing pericentral lobular zone, similar to hepatotoxic substances known to be metabolically toxified by CYP, such as acetaminophen or carbon tetrachloride. To investigate whether CYP influences OTA toxicity, we used a single dose of OTA (7.5 mg/kg; intravenous) with and without pre-treatment with the pan CYP-inhibitor 1-aminobenzotriazole (ABT) 2 h before OTA administration. Blood, urine, as well as liver and kidney tissue samples were collected 24 h after OTA administration for biochemical and histopathological analyses. Inhibition of CYPs by ABT strongly increased the nephro- and hepatotoxicity of OTA. The urinary kidney damage biomarkers kidney injury molecule-1 (KIM-1) and neutrophil gelatinase-associated lipocalin (NGAL) were increased > 126-fold and > 20-fold, respectively, in mice treated with ABT and OTA compared to those receiving OTA alone. The blood biomarkers of liver damage, alanine transaminase (ALT) and aspartate transaminase (AST) both increased > 21- and 30-fold, respectively, when OTA was administered to ABT pre-treated mice compared to the effect of OTA alone. Histological analysis of the liver revealed a pericentral lobular damage induced by OTA despite CYP-inhibition by ABT. Administration of ABT alone caused no hepato- or nephrotoxicity. Overall, the results presented are compatible with a scenario where CYPs mediate the detoxification of OTA, yet the mechanisms responsible for the pericental liver damage pattern still remain to be elucidated.

**Supplementary Information:**

The online version contains supplementary material available at 10.1007/s00204-022-03395-y.

## Introduction

Ochratoxin A (OTA), a mycotoxin produced by several *Aspergillus* and *Penicillium* species, is found worldwide as a contaminant in food and feed commodities (JECFA 2001). It is well absorbed upon ingestion and can be detected in biological samples of exposed humans and other species (EFSA 2020; Malir et al. 2016; Tkaczyk et al. 2021). Exposure to OTA results in a spectrum of dose-related toxic effects, with nephrotoxicity and carcinogenicity considered the most relevant endpoints. The main target organ in both pigs and rodents is the kidney, yet OTA also induces hepatic toxicity in rodents (reviewed by EFSA 2020; Malir et al. 2016; Pfohl-Leszkowicz and Manderville 2007). Chronic OTA administration has been shown to increase the incidence of renal tumors (adenomas/carcinomas) in rats and mice, with male rats being most sensitive; it also increases the incidence of liver tumors in both male and female mice (Bendele et al. 1985; NTP 1989).

Investigations into the mechanisms involved in OTA-induced toxicities have included studies on its accumulation in target organs, biotransformation in several species, and its potential role in oxidative stress (Kőszegi and Poór 2016; Mally and Dekant 2009; Ringot et al. 2006; Tao et al. 2018). Most recently, intravital imaging techniques have been applied to further investigate the subcellular spatio-temporal kinetics of OTA in mice (Ghallab et al. 2021a; Hassan et al. 2022). Other studies have been performed to unravel the mode of action (MoA) of OTA, for example, to determine if it acts by a DNA-reactive MoA (resulting in mutagenicity) or whether perturbations of other critical cellular processes may explain the non-linear dose response observed in organ- and site-specific tumor development (Mally 2012; Tozlovanu et al. 2012). Based on these early studies, induction of apoptosis and autophagy, cell cycle arrest, alterations in the cellular proliferation response and cell signaling, oxidative stress and changes in gene expression have all been proposed as contributing factors.

Despite all the existing studies that aimed to elucidate the underlying mechanism of OTA toxicity, consensus on the primary mechanism of ochratoxicosis has not been reached so far (O'Brien and Dietrich 2005). In particular, controversial views have been expressed on the role of bioactivation of OTA and the relevance of OTA-derived DNA adducts (Manderville 2005; Turesky 2005). In one study, direct genotoxicity (covalent DNA adduct formation) was proposed as a MoA for OTA-mediated carcinogenicity (Tozlovanu et al. 2012), while others who applied more sensitive methodologies found no evidence for OTA-DNA adducts (Delatour et al. 2008; Mally et al. 2005, 2004). In its recent evaluation, the EFSA Panel on contaminants took note of the very low levels of the principle OTA adduct in rat kidney (reported range 20–70 × 10^9^ nucleotides) and stated: “It remains unclear whether and to what extent these DNA adducts are formed in vivo and which metabolic pathway(s) are responsible for the formation” (EFSA 2020).

**Fig. 1:**
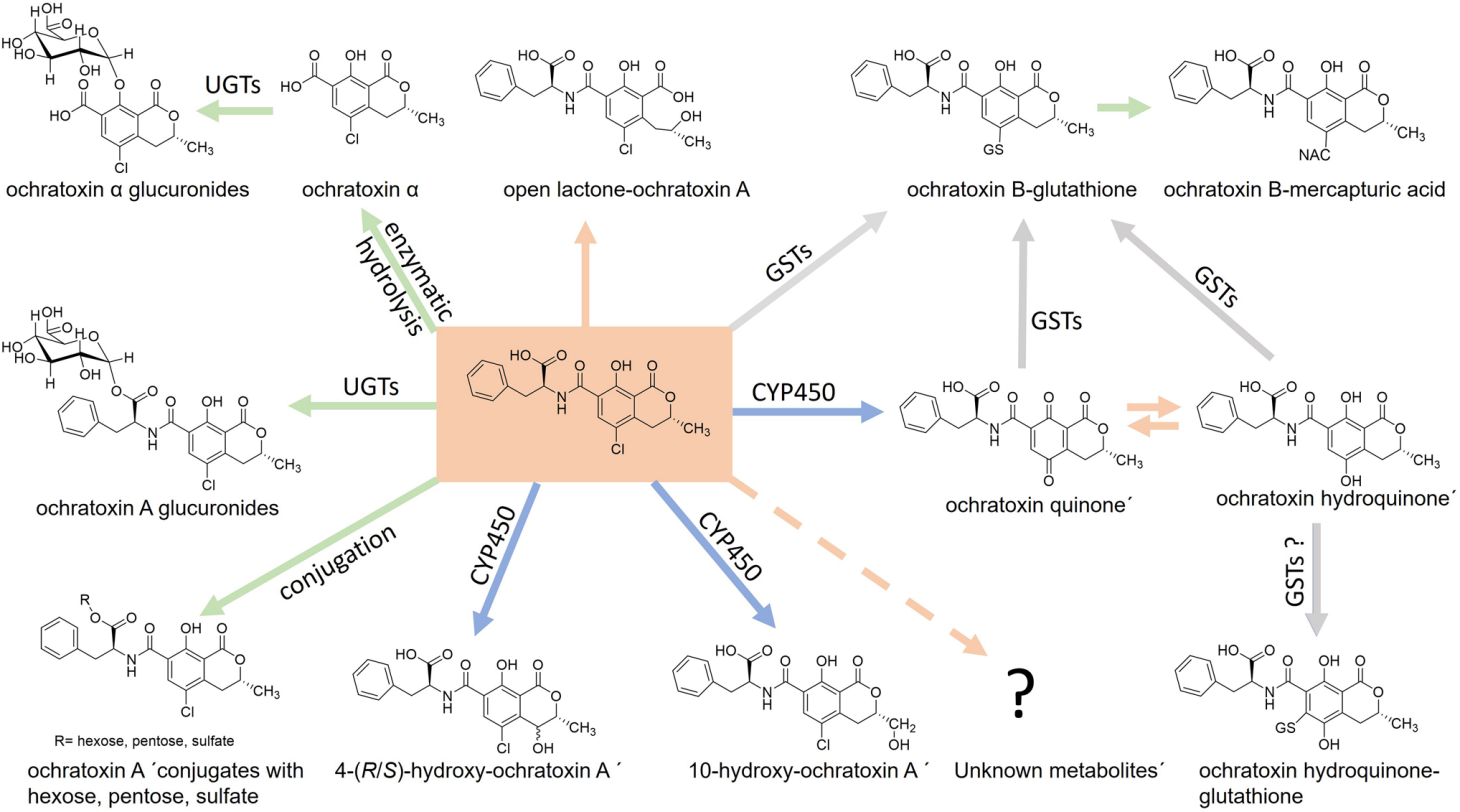
Biotransformation of ochratoxin A (OTA; central box) to metabolites identified in vivo and/or in vitro and proposed intermediates. Some pathways (green arrows) result in the formation of a product (OT-alpha) with relatively low toxicity and to conjugated metabolites which are more readily excreted than the parent mycotoxin. Formation of hydroxylated OTA metabolites by CYP450 enzymes (blue arrows) is also regarded as detoxication reaction. In contrast, CYP-mediated oxidation of OTA to quinone/hydroquinone intermediates (pink arrows) is considered as potential bioactivation reaction. The quinone/hydroquinone couple may react with cellular macromolecules or undergo conjugation by GSTs (grey arrows) to glutathione conjugates, e.g., the OTB-GSH metabolite which is then transformed into a mercapturic acid. Opening of the lactone ring of OTA (pink arrow to the top) yields a product with increased toxicity. See text for further details and references on OTA metabolism. A pink arrow pointing to a question mark denotes hypothetical as yet unknown bioactivation reactions for OTA

As other xenobiotics, OTA undergoes biotransformation reactions catalyzed by phase I and II enzymes: more specifically, OTA is hydrolyzed by mammalian hydrolases and by gut microbiota in animals and in humans (Ringot et al. 2006; Tao et al. 2018). Hydrolysis of OTA to OT-alpha is an important detoxication pathway, as OT-alpha is less toxic and rapidly excreted, mainly as a glucuronide (Fig. 1). Metabolites formed by hydroxylation reactions at the isocoumarin moiety of OTA (4-R/S- and 10-OH-OTA), and by the dechlorination of OTA leading to OTB, appear to represent minor detoxification pathways (EFSA 2020). Of note, these metabolites display lower toxicity in cells and in vivo than the parent mycotoxin itself (Heussner and Bingle 2015). In contrast, the open lactone form of OTA, found in biological samples from various species, can exert similar or higher toxicity than OTA (Dekant et al. 2021; Xiao et al. 1996). Phase II metabolism of OTA leads mainly to glucuronides (Muñoz et al. 2017); other conjugates with sulfate, hexose or pentose have been observed in vitro and in vivo yet only in small amounts (EFSA 2020; Ringot et al. 2006). The recent identification of ochratoxin-*N*-acetyl-l-cysteine (OTB-NAC) in human urines supports previous evidence in animal studies that the mycotoxin can be also converted to glutathione conjugates (Sueck et al. 2020; Tozlovanu et al. 2012). But, so far it remains unknown whether the conjugation reaction between OTA and glutathione (GSH) first requires the formation of a reactive quinone (OTQ) or whether it is directly catalyzed by glutathione-S-transferases (Fig. 1). On the other hand, a recent study found no evidence for formation of OTB-NAC in rats (Dekant et al. 2021). Nevertheless, the detection of an OTA-derived mercapturic acid at significant levels in human urine sheds new light on the metabolism of this mycotoxin (Sueck et al. 2020).

There are still many open questions regarding the metabolism and toxicity of OTA, including whether OTA is activated to electrophilic metabolites that can then bind to DNA, proteins, and other cellular molecules. Proposed pathways for OTA bioactivation and reactive intermediates include (i) an aryl radical formed by reductive dechlorination, (ii) a phenoxyradical formed by one-electron oxidation, and (iii) OTQ formed from OTA as a result of oxidation mediated by peroxidases or cytochrome P450 (CYPs) (Tozlovanu et al. 2012).

In a previous study, we observed that hypoalbuminemia enhanced the uptake of OTA into renal tubular epithelial cells, as well as hepatocytes, and led to increased hepatotoxicity (Hassan et al. 2022). OTA-induced hepatotoxicity showed a pericentral zonation, which is known to occur for substances metabolically activated by cytochrome P450 enzymes that have higher activities in the lobular zone around the central vein (Ghallab et al. 2019). To test whether OTA is metabolically activated by CYPs, we exposed mice to OTA with and without co-exposure to a non-selective pan CYP-inhibitor. Surprisingly, we observed that CYP-inhibition strongly enhanced the hepatotoxicity and nephrotoxicity of OTA; however, pericentral zonation of liver damage occurred despite CYP-inhibition, suggesting that while CYPs do indeed detoxify OTA, other pericentrally located enzymes, which remain to be identified, metabolically activate OTA.

## Materials and methods

### Laboratory animals

C57BL6N 8–10-week-old female and male mice (Janvier Labs, France) were used. The mice were fed ad libitum on a normal diet with free access to water. All experiments were approved by the local authorities (LANUV, North Rhine-Westphalia, Germany, #81–02.04.2020.A304).

### Inhibition of cytochrome P450 enzyme activities by 1-aminobenzotriazole

To block cytochrome P450-dependent metabolism, the mice were treated with 1-aminobenzotriazole (ABT; # J63610.03, ThermoFisher Scientific), a pan inhibitor of cytochrome P450 enzyme activities (de Montellano 2018), at a dose of 150 mg/kg b.w. in phosphate-buffered saline (PBS) orally by gavage. Control mice received only PBS. The application volume of both ABT and PBS was 4 mL/kg b.w. The mice were fasted overnight prior to ABT or PBS applications.

### Induction of acute hepatotoxicity by acetaminophen overdose

To test the efficacy of ABT in cytochrome P450 enzyme activity inhibition, we used the acetaminophen (APAP) model in mice, a standard approach where hepatotoxicity is mediated by cytochrome P450 enzymes (Schneider et al. 2021b; Sezgin et al. 2018). Overnight fasted male C57BL6N mice (*n = *3 mice per group) were treated with PBS or ABT; 2 h later, the mice were challenged with an overdose of APAP (300 mg/kg b.w.; intraperitoneal) that causes acute liver injury (Holland et al. 2022). The mice were fed ad libitum after APAP application. Blood as well as liver tissue samples were collected 6 h after APAP injection for biochemical and histopathological analyses.

### Ochratoxin A application

Overnight fasted female C57BL6/N mice (*n = *5 mice per group) were treated with PBS or ABT; 2 h later, the mice were challenged with ochratoxin A (OTA; 7.5 mg/kg b.w.; intravenous; #10470691, Fisher Scientific). This particular dose of OTA was selected as it was much lower than the LD_50_ (IARC 1993) and similar to doses applied in chronic toxicity studies in mice (Bendele et al. 1985; Kanisawa and Suzuki 1978). Blood as well as liver and kidney tissue samples were collected 24 h after OTA injection for biochemical, immunohistochemical, and histopathological analyses. The blood samples were collected from the heart of anesthetized mice on ethylenediaminetetraacetic acid pre-coated tubes and centrifuged for 10 min at 10,000 RPM for plasma separation. The tissue samples were fixed in 4% paraformaldehyde for 2 days and then further processed and embedded in paraffin (Campos et al. 2020).

### Analysis of blood biomarkers of liver and kidney damage

Biomarkers of liver damage (alanine transaminase and aspartate transaminase), kidney damage (creatinine and blood urea nitrogen), as well as albumin concentrations in mouse plasma were measured using the Piccolo Xpress Chemistry Analyzer (Hitado, Germany). Plasma from control and ochratoxin A-treated mice were measured undiluted. Plasma from ABT plus OTA-treated mice were diluted 1:10 in normal mouse plasma prior to analysis.

### Histopathology and immunohistochemistry analyses

4-µm-thick formalin-fixed paraffin-embedded liver and kidney tissue sections were used for histopathology and immunohistochemistry analyses. All stainings were performed using an autostainer (Discovery Ultra Automated Slide Preparation System, Roche, Germany) based on standard protocols (Ghallab et al. 2021b). For histopathology analysis, hematoxylin and eosin staining was performed. For immunohistochemistry, the following primary antibodies were applied: anti-albumin (# ab192603, Abcam; dilution 1:500), anti-CD45 (#550539, BD Bioscience; dilution 1: 400), and anti-FSP1 (#ab197896, Abcam; dilution 1:200). Appropriate secondary antibodies were used (Hassan et al. 2022).

### Analysis of neutrophil gelatinase-associated lipocalin (NGAL) in mouse urine

Concentrations of NGAL/Lcn-2 were determined in urine samples using the mouse lipocalin-2/NGAL DuoSet ELISA kit plus the DuoSet ELISA Ancillary Reagent Kit 2 from R&D systems (DY1857 and DY008), according to manufacturer’s protocol. The urine samples were collected 24 h after OTA application by cannulation of the urinary bladder using a 26-gauge cannula (SAI-infusion Technologies) as previously described (Reis et al. 2011). A 96-well plate was coated with 4 µg/mL of rat anti-mouse lipocalin-2 capture antibody and incubated overnight at room temperature. The next day, the blocking solution was added after a washing step and incubated for 1 h. Then, the previously diluted urine samples (in 1% BSA in PBS) and recombinant NGAL standards were added to the coated wells and incubated for 2 h. Biotinylated rat anti-mouse lipocalin-2 detection antibody was added after a washing step and incubated for 2 h. Subsequently, the antibody-NGAL sandwich complex was monitored by streptavidin conjugated to horseradish peroxidase (HRP). Finally, the optical density of the color-forming TMB substrate was measured at an optical density of 450 nm using a microplate reader (Infinite M200 Pro, Tecan). The concentration of NGAL/Lcn-2 of each sample was calculated from the standard curve.

### Analysis of kidney injury molecule-1 (KIM-1) in mouse urine

Among a panel of urinary biomarkers, KIM-1 has been shown to be best suited for detecting OTA-induced nephrotoxicity (Hoffmann et al. 2010). Concentrations of KIM-1 were determined in urine using the Mouse KIM 1 ELISA Kit from Abcam (ab213477) according to manufacturer’s protocol. Briefly, the previously diluted urine samples and mouse recombinant KIM-1 standards were added to the wells followed by the antibody cocktail (capture and detector antibodies) and incubated for 1 h at room temperature on a plate shaker. After the incubation, the antibody-KIM-1 sandwich complex was monitored by streptavidin conjugated to horseradish peroxidase (HRP). Finally, the optical density of the color-forming TMB substrate was measured at an optical density of 450 nm using a microplate reader (Infinite M200 Pro, Tecan) and the concentration of KIM-1 of each sample was calculated from the standard curve.

### Intravital imaging

To test if ABT treatment influences OTA transport kinetics, intravital imaging of OTA transport in the livers of mice pre-treated with PBS or ABT was performed using a two-photon microscope (LSM MP7, Zeiss, Germany), as previously described (Hassan 2016; Hassan et al. 2022; Remetic et al. 2022). Tetramethylrhodamine-ethyl ester (TMRE; #T669, ThermoFisher Scientific) was injected prior to the recording to visualize hepatocyte morphology and lobular zonation (Ghallab et al. 2022). OTA was applied as a bolus (7.5 mg/kg b.w. intravenous) within a few seconds after the start of recordings using a tail vein catheter (SAI-infusion, IL, USA). Approximately 5 min before the end of recording, a bolus of the bile acid analogue cholyl-lysyl-fluorescein (CLF) was administered to allow detection of the position of the bile canaliculi (Schneider et al. 2021a). Three mice were imaged per group and representative videos are shown in “Results”.

### Image analysis

As a pre-processing step of the image quantification procedure, rigid body registration was performed using StackReg (Thévenaz et al. 1998) to compensate for tissue motion (e.g., due to respiration and heartbeat) in the recorded time series. Two-dimensional projections were created from these stabilized videos using average and maximum operators. For the segmentation of tissue compartments in these 2D projections, the auto-context segmentation workflow of the interactive image segmentation software ilastik (version 1.3.3post1) was used (Berg et al. 2019). The compartments considered included blood sinusoids, hepatocellular cytoplasm, and bile canaliculi. Mean raw OTA intensities were measured per compartment and frame.

### Statistical analysis

The data were analyzed for statistical significance using GraphPad Prism version 9.3.1 (GraphPad Software, Inc., La Jolla, CA). *p* value ≤ 0.05 was considered statistically significant. The applied statistical tests are indicated in the respective figure legends.

## Results

### Aminobenzotriazole blocks the hepatotoxicity of acetaminophen (APAP)

**Fig. 2:**
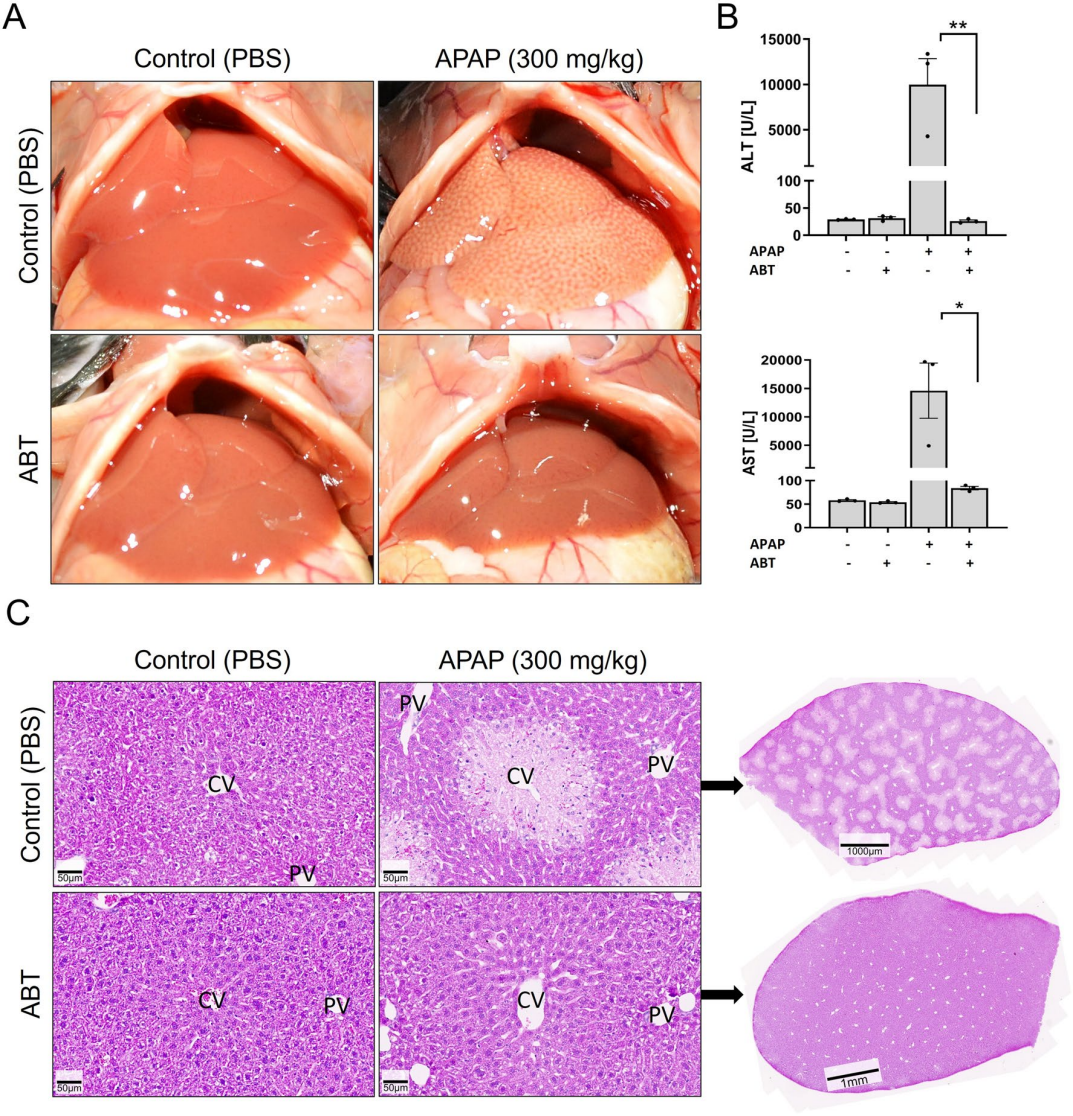
CYP-inhibition by aminobenzotriazole (ABT) blocks acetaminophen (APAP) induced hepatotoxicity. **A** Macroscopical appearance of livers 6 h after an overdose of 300 mg/kg APAP alone or in combination with the non-specific CYP-inhibitor ABT (150 mg/kg; gavage). ABT was administered 2 h before APAP. The control groups received only vehicle (PBS) or ABT. **B** Plasma activities of the liver enzyme ALT and AST 6 h after APAP administration with and without ABT compared to vehicle or ABT controls; *p value ≤ 0.05, **p value ≤ 0.01 Tukey’s multiple comparisons test; *n = *3 mice per group. **C** Histological appearance (H&E staining) of control (vehicle or ABT treated) and APAP-treated livers with and without ABT in 20-fold magnification with accompanying overview of entire liver lobules

To validate if the non-selective inhibitor of cytochrome P450 enzymes, aminobenzotriazole (ABT) can block CYP-mediated hepatotoxicity under the here-applied conditions, acetaminophen (APAP) was used as a positive control. A dose of 150 mg/kg ABT was given by gavage 2 h before i.p. injection of 300 mg/kg APAP (APAP). When given as a single substance, APAP induced the typical pattern of macroscopically visible white spots on the liver surface that is due to pericentral necrosis (Fig. 2A). In contrast, the liver tissue did not differ from vehicle controls when ABT was administered before APAP intoxication. Activities of the liver enzymes, ALT and AST showed massive elevation in the blood upon APAP overdose (Fig. 2B). ABT completely blocked this increase so that ALT and AST activities remained similar to control levels. ABT alone did not cause any increase in serum liver enzyme activities or histological alterations. Histological examination showed pericentral dead cell regions after APAP overdose that were completely prevented by pre-treatment with ABT (Fig. 2C). Thus, ABT efficiently blocked CYP-mediated metabolic activation under the here-applied experimental conditions.

### CYP-inhibition strongly enhances pericentral ochratoxin A induced hepatotoxicity

**Fig. 3:**
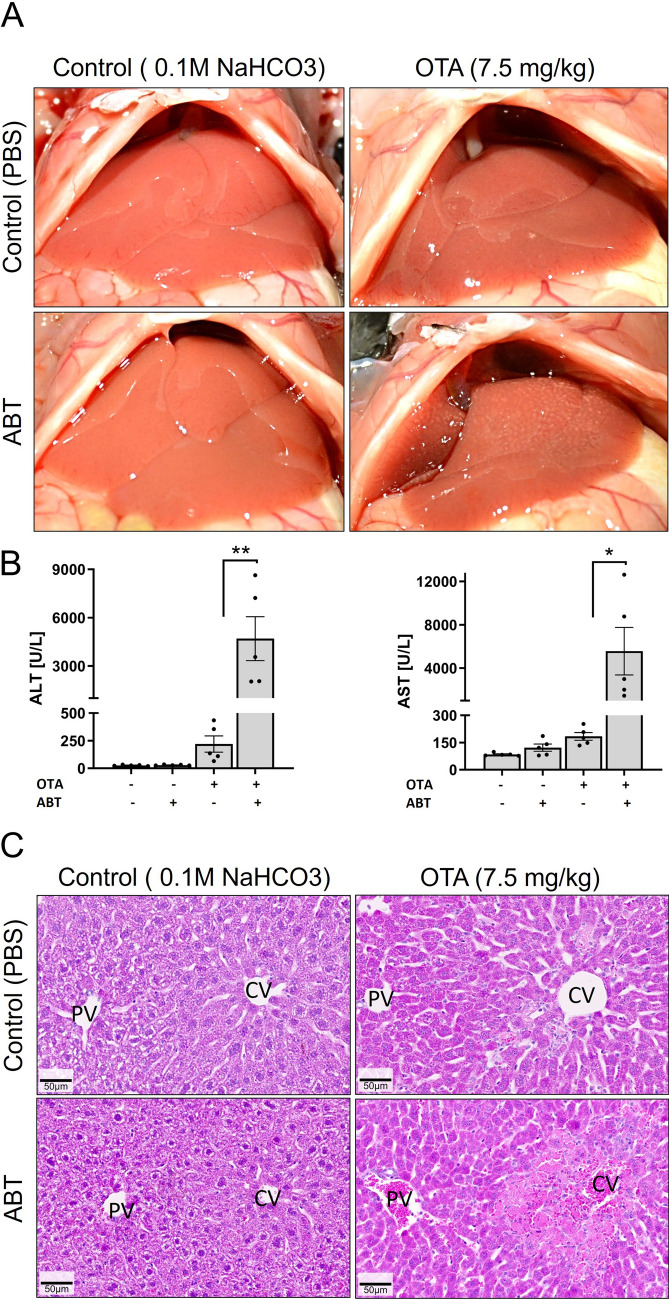
CYP-inhibition enhances the hepatotoxicity of ochratoxin A (OTA). **A** Macroscopic appearance of livers 24 h after administration of OTA (7.5 mg/kg; i.v.) with and without ABT (150 mg/kg, gavage; administered 2 h before OTA) compared to vehicle (PBS) or ABT controls. **B** Plasma activities of liver enzymes 24 h after OTA administration with and without ABT compared to vehicle or ABT controls; *p value ≤ 0.05, **p value ≤ 0.01 Tukey’s multiple comparisons test; *n = *5 mice per group. **C** Histological appearance in H&E-stained tissue sections; scale bars: 50 µm

Next, we tested the hepatotoxicity of ochratoxin A (OTA) with and without prior administration of ABT under the same conditions established above for APAP. For this purpose, a dose of 7.5 mg/kg OTA (administered intravenously) was chosen. OTA alone did not cause any change in the macroscopic appearance of the liver (Fig. 3A). In combination with ABT, OTA led to a dotted pattern on the surface of the liver, although this phenomenon was less pronounced compared to APAP. OTA alone induced a slight elevation in serum ALT and AST levels (Fig. 3B), which dramatically increased (> 21-fold) in combination with ABT. Histological analysis showed single necrotic cells or small necrotic cell clusters in the pericentral region of livers exposed to OTA alone (Fig. 3C). Upon co-administration of ABT and OTA, large pericentral necrotic dead cell areas with hemorrhage were observed (Fig. 3C). Thus, non-selective inhibition of CYPs strongly enhanced the hepatotoxicity of OTA.

**Fig. 4:**
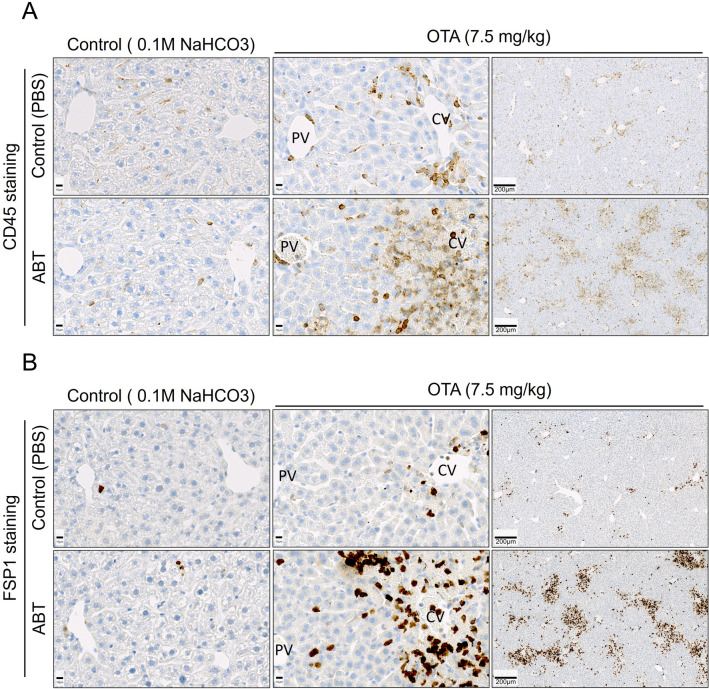
Infiltration of immune cells into regions of pericentral tissue damage induced by OTA intoxication with and without ABT treatment compared to vehicle or ABT controls. **A** Immunostaining for the common leukocyte antigen CD45 and the macrophage marker FSP1 (**B**). The same experimental design as described in Fig. 3 was applied; scale bars: 10 µm (closeup) and 200 µm (overview)

Usually, liver injury is accompanied by the infiltration of immune cells into damaged tissue. Therefore, we immunostained liver tissue sections with the common leukocyte antigen, CD45, and with FSP1, a marker of a specific subpopulation of inflammatory macrophages (Fig. 4A, B). While OTA alone induced a moderate increase in both CD45 and FSP1, the combination of OTA with ABT led to a massive increase in immune cell infiltration into the pericentral regions of the liver lobules. Thus, the immunostaining data of leukocytes and macrophages corresponded well with the clinical chemistry and H&E staining results, illustrating that CYP-inhibition substantially aggravated OTA-induced liver damage.

### CYP-inhibition enhances OTA-mediated nephrotoxicity

**Fig. 5:**
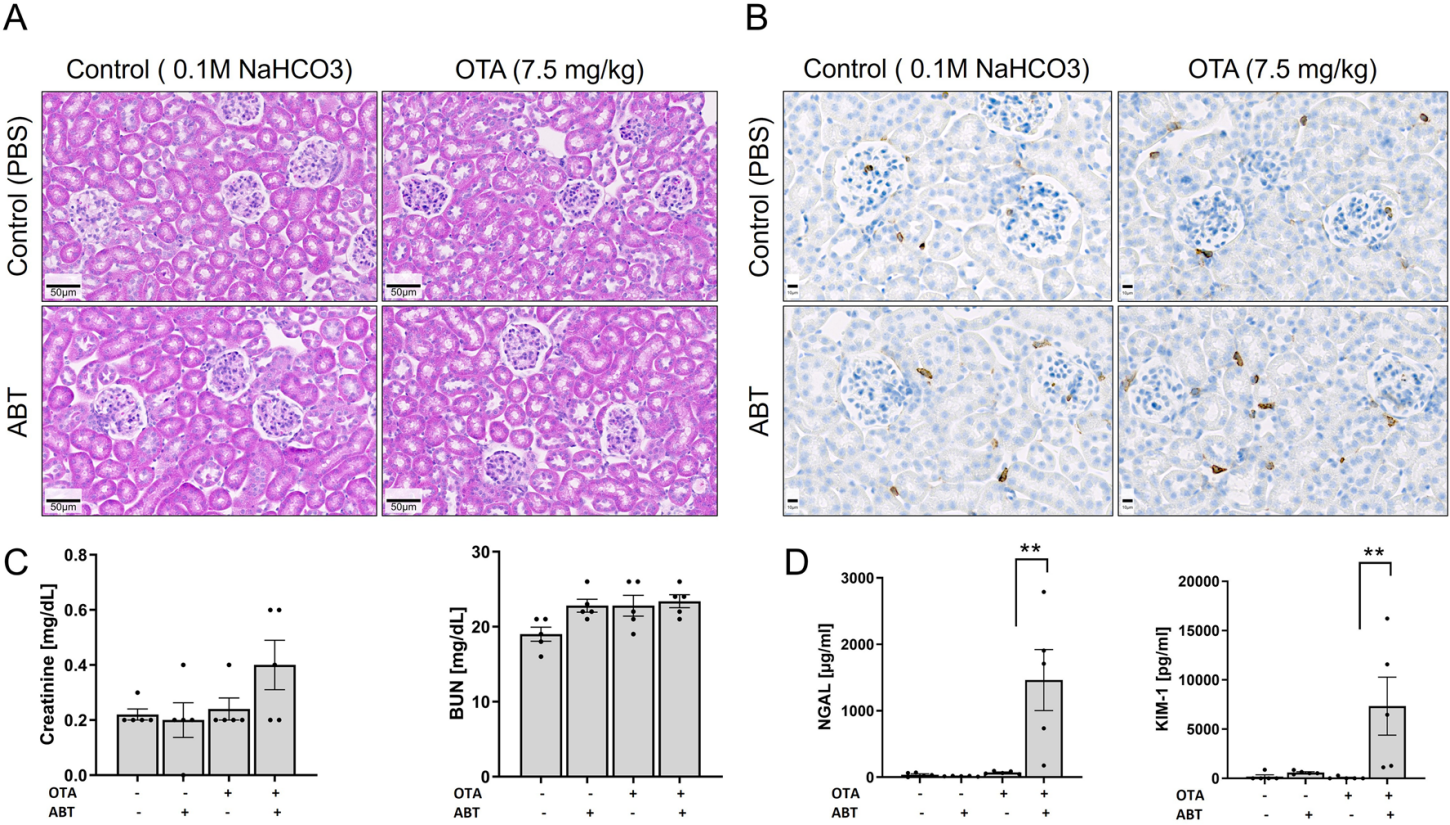
CYP-inhibition enhances the nephrotoxicity of OTA. **A** H&E staining; scale bars 50 µm. **B** CD45 immunostaining; scale bars 10 µm. **C** Serum creatinine and blood urea nitrogen. **D** NGAL and KIM-1 urinary concentrations 24 h after OTA administration with and without ABT compared to vehicle or ABT controls; **p value ≤ 0.01 Tukey’s multiple comparisons test; *n = *5 mice per group. The same experimental design as in Fig. 3 was applied

The same mice that were analyzed above for liver injury were also used to study possible nephrotoxic effects of OTA with and without CYPs inhibition. H&E staining and immunostaining for CD45 did not reveal any differences between OTA-treated and control mice 24 h after administration, nor did the combination of OTA and ABT lead to any obvious histological changes (Fig. 5A, B). Serum creatinine levels showed a small, but statistically non-significant increase in the combined OTA plus ABT group compared to OTA alone (Fig. 5C), while no changes were observed in blood urea nitrogen (BUN) in any of the treatment groups compared to control mice (Fig. 5C). However, levels of neutrophil gelatinase-associated lipocalin (NGAL) and of kidney injury molecule-1 (KIM-1), a more sensitive marker of kidney damage, showed a more than 20-fold and 126-fold increase, respectively, in the OTA plus ABT group compared to OTA alone (Fig. 5D), which had no significant effect on NGAL or KIM-1 levels compared to the vehicle control.

### No alterations in albumin and OTA kinetics due to ABT

**Fig. 6:**
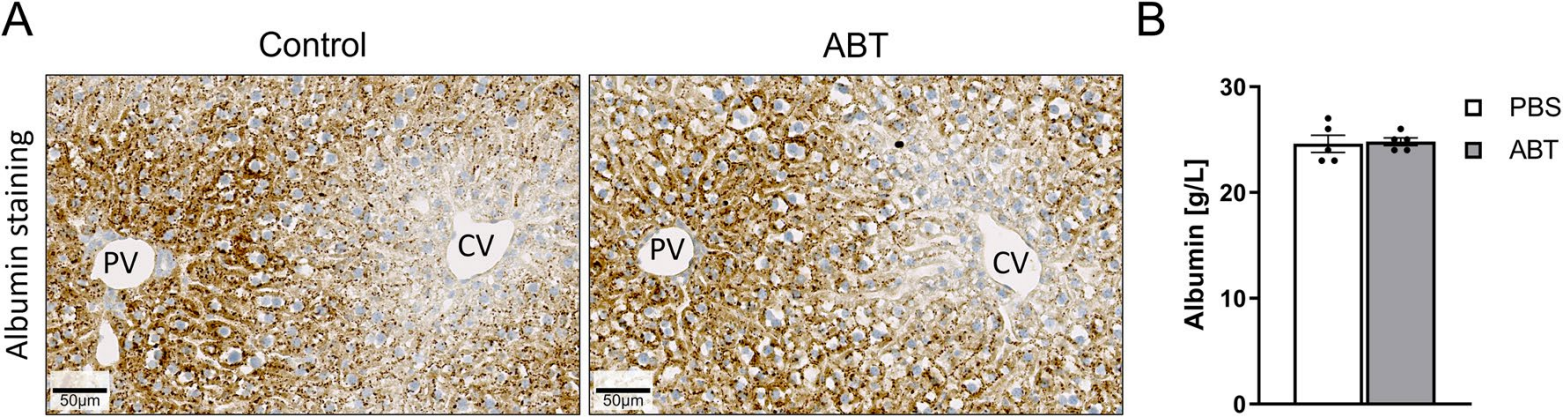
Treatment with ABT does not influence albumin expression in the liver as evidenced by immunostaining (**A**) and serum albumin (**B**); scale bars 50 µm; *n = *5 mice per group. The mice received a dose of 150 mg/kg ABT by gavage and the analyses were performed 24 h later

**Fig. 7:**
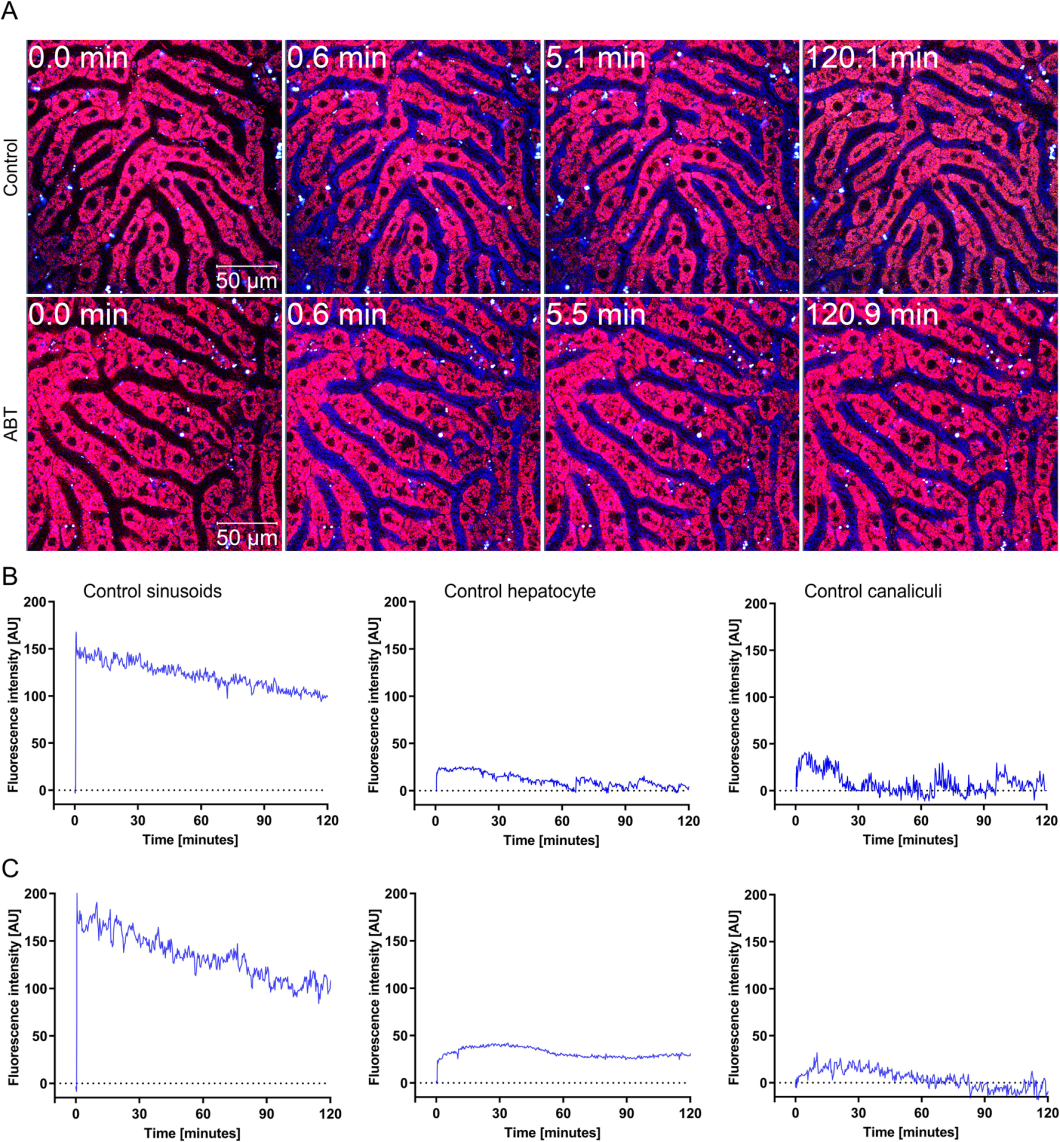
Treatment with ABT has no major influence on the transport of OTA from the sinusoidal blood into hepatocytes and secretion into the bile canaliculi. **A** Intravital imaging of OTA (7.5 mg/kg intravenous) transport in livers of mice pre-treated (2 h) with ABT (150 mg/kg, gavage) or vehicle. The time in the upper left gives the minutes after injection of OTA. OTA is visualized by its blue auto-fluorescence; the morphology of hepatocytes is visualized by the red dye TMRE. **B** and **C** Quantification of OTA-associated fluorescence in blood sinusoids, hepatocytes and in bile canaliculi in controls (vehicle) and in ABT pre-treated mice. The corresponding time-lapse videos are available in the supplement. Three mice were imaged per group

Recently, it was shown that hypoalbuminemia increases the susceptibility of OTA-mediated hepatotoxicity in mice (Hassan et al. 2022). Therefore, we controlled if ABT under conditions used in the present study influenced albumin in serum or in liver tissue, because this mechanism may enhance OTA toxicity. However, no difference in albumin was observed in immunostained liver tissue sections 24 h after administration of 150 mg/kg ABT (Fig. 6A), nor did ABT influence the concentrations of serum albumin (Fig. 6B). Recently, intravital imaging techniques were established that allow the label-free analysis of the transport of OTA from liver sinusoidal blood into hepatocytes (Fig. 7; Supplement Video 1). Therefore, to study if ABT influences the uptake kinetics of OTA from sinusoidal blood into hepatocytes, intravital two-photon imaging was performed. Under control conditions, the OTA-associated blue auto-fluorescence showed a sharp increase in the sinusoidal blood within seconds after intravenous injection, followed by a slow decrease (Supplement Video 1A; Fig. 7B). After the increase in the sinusoids, OTA-associated fluorescence showed an increase in the hepatocytes and in bile canaliculi. At the end of the analysis period, an intravenous bolus injection of the green-fluorescent bile salt analogue cholyl-lysyl-fluorescein (CLF) was performed as a positive control as it is known to be rapidly taken up into hepatocytes and excreted into bile canaliculi (Vartak et al. 2021) (Supplement Video 1A). To determine if ABT influences the transport kinetics of OTA, the same intravital experiment was performed after pre-treatment with ABT (Supplement Video 1B; Fig. 7C). To allow a quantitative comparison, an artificial intelligence-based method was applied to segment the sinusoids, hepatocytes and bile canaliculi, and to quantify the OTA-associated blue fluorescence in these compartments. This analysis revealed no major differences in the OTA-signal between ABT pre-treatment and vehicle controls, neither in the sinusoids, nor in hepatocytes or bile canaliculi. It should be considered that the detected blue fluorescence may be due to the parent compound OTA or its metabolites. Nevertheless, the lack of any major difference with and without ABT suggests that the CYP-inhibitor did not influence the kinetics of OTA uptake into the hepatocytes.

## Discussion

In the present study, we observed that non-selective inhibition of cytochrome P450 strongly enhanced the hepatotoxicity and nephrotoxicity of OTA. This result was surprising since we initially hypothesized that OTA may be metabolically activated by CYPs because of the previously observed hepatotoxicity specifically in the cytochrome P450 positive pericentral lobular zone (Atroshi et al. 2000; Hassan et al. 2022). This typical pericentral damage pattern is known from other compounds that are metabolically activated by cytochrome P450, such as APAP (Sezgin et al. 2018) and CCl_4_ (Ghallab et al. 2016; Schenk et al. 2017).

The size of the effect of the non-selective CYP-inhibitor ABT (de Montellano 2018) was remarkable; doses of OTA that, as a single compound, only induced a very weak increase in serum liver enzyme activities, caused a dramatic increase when OTA was combined with the CYP-inhibitor. The effect was similarly considerable in the kidney, where OTA alone elicited no effect on NGAL, a marker of kidney damage, but in combination with ABT resulted in a more than 20-fold increase in NGAL levels compared to control values. While it was clearly demonstrated in this study that cytochrome P450 enzymes reduce the hepatotoxicity and nephrotoxicity of OTA, the involved detoxification pathways still remain unknown. The aforementioned hydrolysis which may be followed by glucuronidation (Fig. 1) is a likely candidate. Furthermore, the above-mentioned formation of a reactive quinone followed by GSH detoxification seems also plausible. Moreover, it remains elusive why OTA causes cytotoxicity specifically in the pericentral lobular zone that also expresses much higher levels of CYPs than the midzonal and periportal lobular regions (Ghallab et al. 2019). This phenomenon was observed as mild hepatotoxicity with single dispersed hepatocyte death events in the pericentral region for the single compound OTA, and as massive pericentral dead cell areas for the combined administration of OTA plus ABT. One possible explanation could be that enzymes other than CYPs, which are preferentially expressed in the pericentral zone, e.g., flavin-containing monooxygenases (FMOs) (Başaran and Can Eke 2017; Novick et al. 2009), are responsible for the metabolic activation of OTA to a toxic species. Alternatively, the parent compound OTA could be responsible for the pericentral zonation of damage, if detoxifying enzymes would be preferentially expressed in the periportal lobular zone could be. Thus, the mechanism responsible for the pericentral damage pattern of OTA still remains to be elucidated. To our knowledge this is the first case where a pericentral damage pattern of hepatotoxicity mediated by other factors than cytochrome P450 was observed.

In addition to the mechanisms proposed above, an alternative explanation was that ABT decreases serum albumin concentrations, as well as concentrations of albumin in hepatocytes. Recently, we used albumin knockout mice and demonstrated that a higher fraction of OTA passes from the blood into hepatocytes and renal tubular epithelial cells resulting in increased levels of toxicity in hypoalbuminemic compared to wild-type mice (Hassan et al. 2022). However, the here-performed experiments demonstrated that ABT did not decrease albumin levels, neither in serum nor in hepatocytes, indicating that ABT does not exacerbate OTA toxicity by reducing albumin levels. A further alternative explanation was that ABT alters the toxicokinetics of OTA. However, intravital imaging demonstrated that ABT had no influence on the transport kinetics of OTA from the sinusoidal blood into hepatocytes and subsequent secretion into bile canaliculi. It should be mentioned that metabolites of OTA may also exhibit fluorescence but this is not relevant for the conclusions of the present study that ABT did not influence the uptake of OTA from sinusoidal blood into hepatocytes.

Our analysis revealed zonated expression of albumin in hepatocytes with higher levels in the periportal and lower levels in the pericentral compartment of the liver lobules. Based on this, one may hypothesize that the zonated pericentral damage of OTA is due to lower expression of albumin in the pericentral hepatocytes as evidenced by immunostaining in the present study. However, intoxication of homozygous albumin knockout mice with OTA also revealed a pericentral damage pattern (Hassan et al. 2022). Therefore, the pericentral zonation of OTA-induced liver damage must be independent of the zonated expression of albumin. For further understanding of the underlying mechanisms, analysis of tissues and fluids for OTA and the known metabolites could be a promising next step.

It should be considered that we administered OTA intravenously, because this route of administration allows the intravital analysis of tissue concentrations by two-photon imaging. The selected single dose of 7.5 mg/kg is much lower than the median lethal dose of OTA (IARC 1993), which offers good conditions to detect an increased effect due to CYP-inhibition.

In conclusion, the present study demonstrated that CYP-inhibition massively increases the hepato- as well as nephrotoxicity of OTA, and that a pericentral damage pattern is induced in the liver.

## Supplementary Information

Below is the link to the electronic supplementary material.

Supplementary file1 Supplemental Video 1: Transport of OTA (blue auto-fluorescence) from the blood sinusoids of the liver into hepatocytes and secretion into bile canaliculi in vehicle controls (A) and in mice that received 150 mg/kg ABT two hours before injection of OTA (B). The time after injection of OTA is given in the upper left corner. The morphology of hepatocytes is visualized by the red fluorescent mitochondrial dye TMRE. The videos correspond to the stills and image analyses shown in Fig. 7. (M4V 39312 kb)

Supplementary file2 (MP4 39555 kb)

## Abbreviations

ABT: Aminobenzotriazole
ALT: Alanine transaminase
APAP: Acetaminophen
AST: Aspartate transaminase
BUN: Blood urea nitrogen
CYPs: Cytochrome P450
CCl_4_: Carbon tetrachloride
CLF: Cholyl-lysyl-fluorescein
KIM-1: Kidney injury molecule-1
NGAL: Neutrophil gelatinase-associated lipocalin
TMRE: Tetramethylrhodamine-ethyl ester
OTA: Ochratoxin A
